# Effects of climate change and pollen supplementation on the reproductive success of two grassland plant species

**DOI:** 10.1002/ece3.8501

**Published:** 2022-01-24

**Authors:** Martin Andrzejak, Lotte Korell, Harald Auge, Tiffany M. Knight

**Affiliations:** ^1^ Department of Community Ecology Helmholtz Centre for Environmental Research GmbH – UFZ Halle (Saale) Germany; ^2^ Institute of Biology Martin Luther University Halle‐Wittenberg Halle (Saale) Germany; ^3^ German Centre for Integrative Biodiversity Research (iDiv) Halle‐Jena‐Leipzig Leipzig Germany

**Keywords:** climate change, pollen supplementation, pollination

## Abstract

Climate change has the potential to alter plant reproductive success directly and indirectly through disruptions in animal pollination. Climate models project altered seasonal precipitation patterns, and thus, the effects of climate change on available resources and pollination services will depend on the season. Plants have evolved reproductive strategies to so they are not limited by either pollen or water availability in their reproductive success, and therefore, we expect that the disruption of climate change might cause plants to be more pollen limited in seasons that become wetter than they were historically. In this study, we conducted a pollen supplementation experiment within the Global Change Experiment Facility (GCEF) in Central Germany. The GCEF experimentally manipulates future climate based on a realistic scenario of climate change for the region (drier summers and wetter springs and falls) in a native grassland ecosystem. We quantified seed production of two perennial species *Dianthus carthusianorum* and *Scabiosa ochroleuca* in response to pollination treatments (control, supplement), climate treatments (ambient and future) and season (summer and fall). *Dianthus carthusianorum* produced more seeds in future climate conditions independent of the season, but only when given supplemental pollen. Both species showed an increased reproduction in summer compared with the fall. We did not find evidence for our specific expectation of higher pollen limitation in the future climate and fall season (i.e., no three‐way interaction pollination × season × climate), which might be explained by the high‐drought tolerance and generalized pollination of our focal plant species. We conclude that plant reproductive success has the potential to change with changing climates and that this change will depend on how pollinator services change in the future. We offer many suggestions for future studies that are necessary to understand the context dependence and underlying mechanisms of plant reproductive responses to climate.

## Graphical abstract

In this study, we conduct pollen supplementation experiments on two plant species within a climate change experiment in which precipitation is manipulated in the context of a regional climate change scenario. *Dianthus carthusianorum* produced more seeds in future climate conditions independent of the season, but only when given supplemental pollen. Both species showed an increased reproduction in summer compared with the fall, but we did not find any evidence for our expectation of higher pollen limitation in the future climate and fall season.

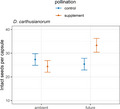

## 1 INTRODUCTION

Climate change may influence plant populations directly, by affecting their fitness, or indirectly via changes in biotic interactions. For example, climate change can directly alter plant reproduction (Eckert et al., 2010; Hedhly et al., 2009) by changing the resources available (e.g., resources needed to make flowers, fruits, and seeds; Koti et al., 2005; Takkis et al., 2015). In temperate grassland ecosystems, water is the most limiting abiotic factor for plant fitness (Lambers & Oliveira, 2008), and climate change is projected to change precipitation patterns. These changes in precipitation will be heterogeneous in space and time, depending on the region of the world and the season (Dore, 2005; Hundecha & Bárdossy, 2005; IPCC, 2014; Kudo & Cooper, 2019; Stocker et al., 2013). In Central Germany, for example, climate models project dryer summers and wetter falls in the future (Döscher et al., 2002; Jacob & Podzun, 1997; Rockel et al., 2008; Wagner et al., 2013). Climate change can indirectly influence plant reproduction by altering the services provided by animal pollinators, which 87.5% of flowering plants rely on for reproduction (Ollerton et al., 2011). For instance, climate change has been linked to declines in the abundance and diversity of pollinators and shifts in their flight periods (Giannini et al., 2017; Knight et al., 2018; Scaven & Rafferty, 2013; Settele et al., 2016).

Multiple limitation theory predicts that plants evolve to be equally limited by pollen and resources (e.g., water; Haig & Westoby, 1988). Their evolved traits for a given environment should result in plants spending an optimal amount of resources on attracting pollinators to maximize seed production, which also requires resources for seed maturation. Thus, increases in precipitation (and therefore in the resource water) might cause plant reproduction to become more limited by pollen receipt, if pollinators cannot provide sufficient pollination for the resources that are potentially available. Decreases in precipitation might, in turn, cause plant reproduction to become more resource limited, and therefore, adding more pollen should not increase plant reproductive success. Thus, we would at extreme precipitation events and oversaturation expect a positive relationship between precipitation and pollen limitation, and a positive but saturating relationship between precipitation and offspring production. However, we acknowledge that extremely high precipitation might physiologically stress plants, causing the relationship between precipitation and offspring production to be a hump‐shaped function, where the increase in water resources leads to a decrease in the number or quality of offspring at high precipitation values. Pollen supplementation experiments, which measure how plant reproduction responds to experimental pollen addition, often find that plants are significantly pollen limited (Bennett et al., 2020; Burd, 1994; Knight et al., 2005; Larson & Barrett, 2000). This might be due to natural variation in resources and pollen across space and time or by anthropogenic factors, such as climate change, that push plants away from their evolved optima (Ashman et al., 2004; Bennett et al., 2020; Knight et al., 2018). However, to our knowledge there exists no study that has quantified pollen limitation in the context of changing climate (and thus resources availability) manipulating resources based on regionally realistic climate change scenarios projected by (regional) climate models (Korell et al., 2020).

While understanding how resources and pollen influence plant reproductive success has a long history (Bierzychudek, 1981; Haig & Westoby, 1988), this topic is increasingly relevant to understanding and predicting plant responses to climate change. A few studies have factorially manipulated resources and pollen (Brookes & Jesson, 2007; Campbell & Halama, 2012; Ne’eman et al., 2006). These studies expected that these experimental treatments would interactively influence plant reproductive success, with the greatest levels of pollen limitation for the resource addition treatment. However, these factorial studies did not find evidence for significant interactions (Brookes & Jesson, 2007; Campbell & Halama, 2012; Ne’eman et al., 2006). Further, the resource manipulations were not in the context of realistic climate change scenarios. At this time, we do not have a synthetic understanding of how pollen and resources jointly influence plant reproductive success, due to the small number of empirical studies, and the heterogeneity across these studies in the type of resource considered and the methods of resource manipulation.

In this study, we conduct pollen supplementation experiments within a climate change experiment in which precipitation is manipulated in the context of a regional climate change scenario. We quantify how precipitation change, which varies across seasons, influences the magnitude of pollen limitation and plant reproductive success. The experimental climate treatments applied are specific to the study region, which includes dryer summers under future climate conditions and wetter falls. For our pollinator‐dependent plant species, we expect pollen supplementation will increase reproductive success; however, the magnitude of that increase should depend on climate (ambient and future) and season (summer and fall)—with pollen limitation being highest in the future climate during the fall season (i.e., the treatment combination that is associated with the highest precipitation).

## 2 METHODS

### 2.1 Study species

In order for us to quantify the influence of climate change and pollination on the reproduction of plants, we needed to select the study species that:

i) Were highly abundant so that a minimum number of eight individuals per plot could be used in the experiment, for the study along with additional individuals that could serve as pollen donors for the pollen supplementation treatment.
ii) Rely on animal pollinators to reproduce, as we were interested in pollen limitation and thus have the potential to suffer from pollen limitation.

Two species fit these criteria, and *Dianthus carthusianorum* L. and *Scabiosa ochroleuca* L. are perennial herbaceous species in the families Caryophyllaceae and Dipsacaceae, respectively. Both species are native to Europe, drought resistant, and adapted to nutrient‐poor habitats. Both species depend on insect pollination for sexual reproduction (Klotz et al., 2002). *Dianthus carthusianorum* generally avoids self‐pollination by protandry, but in some cases, selfing is known to occur at the end of the lifecycle of a flower (Bloch et al., 2006). *Dianthus carthusianorum and S*. *ochroleuca* can reproduce asexually with clones (Hensen, 1997). Further, *D*. *carthusianorum* is listed as vulnerable at the German red list and *S*. *ochroleuca* has the status as endangered in the German red list (https://www.rote‐liste‐zentrum.de); neither species has been evaluated by the IUCN red list. Because we found conflicting information about the pollinator dependence for these species (Bloch et al., 2006; Klotz et al., 2002), we established a pollinator exclusion experiment to directly measure pollinator dependence (Appendix 1). Plants with experimentally bagged flowers (pollinator exclusion) had significantly fewer intact seeds compared with plants in the control and pollen supplement treatments (see description below), suggesting that both plant species depend on pollinators for their reproduction (Appendix 2).

### 2.2 Study system

We conducted the study at the Global Change Experimental Facility (GCEF), which is part of the field research station of the Helmholtz Centre for Environmental Research GmbH—UFZ at Bad Lauchstädt (51°22060 N, 11°50060 E, 118 m a.s.l.) and was established in 2013. It is a unique field experiment, designed to answer questions about the influence of climate change on different land‐use systems (Appendix 3), including extensively used meadows. The experiment is a split‐plot design with climate (ambient and future) as the main plot factor and land use (Appendix 3) as the subplot factor (Schädler et al., 2019). Each main plot (80 × 24 m) is divided into five subplots (16 × 24 m) that are randomly assigned to one of five land‐use treatments. For our study, we focused on the extensively used meadows, resulting in 10 plots we could collect samples from (five ambient and five future climate plots). Each main plot is entirely covered by a tent‐like steel construction holding a plastic roof and a plastic sidewall at the eastern and western end of the future main plots. The closable roof of the future main plots enables the manipulation of climate (see Schädler et al., 2019 for details), which roofs will only close, along with the sidewalls, when it is raining and at night (for a temperature increase) and otherwise stay open. The closed roofs should thus not represent an obstacle for the pollinating insects of our study species as they would not fly during the night or are diurnal and do not fly in the rain. This ensures that these barriers are only present when potential pollinators of the study species are not flying, minimizing the impact they potentially have on their foraging decisions. To ensure comparability between ambient and future climate main plots, the basic infrastructure was built around ambient main plots as well, missing the roof and sidewall feature of the future main plots. The climate manipulation is based on a mean future climate projected for the period of 2070–2100 from 12 climate simulations specifically for that region (www.regionaler‐klimaatlas.de; see details Schädler et al., 2019). In the “mean climate” scenario, the change in precipitation depends on the season. Therefore, the precipitation is decreased by 20% in summer and increased by 10% in spring and fall in the future climate treatment. The soil of the study system is considered nutrient rich (Haplic Chernozem.) and therefore should provide adequate nutrient resources for our study species (Schädler et al., 2019).

The extensively used meadow contains typical grassland plant species that are also found in the natural habitats surrounding the GCEF. The seeds used originate from the local species pool, and seeds were collected from many individuals and several local populations, if possible, to ensure that adequate genetic variation was present (Madaj et al., 2020). More than 50 different species were sown in the habitat in early spring 2014. The extensively used management strategy represents an example for sustainable grassland management while maintaining biodiversity (Schädler et al., 2019). Specifically, habitats in this treatment are mown twice a year (mid‐ to late spring and mid of summer). Depending on the growth of the vegetation, mowing can be reduced to just once in mid to late spring. For a more detailed overview about the GCEF, see Schädler et al., 2019.

### 2.3 Experimental design

**FIGURE 1:**
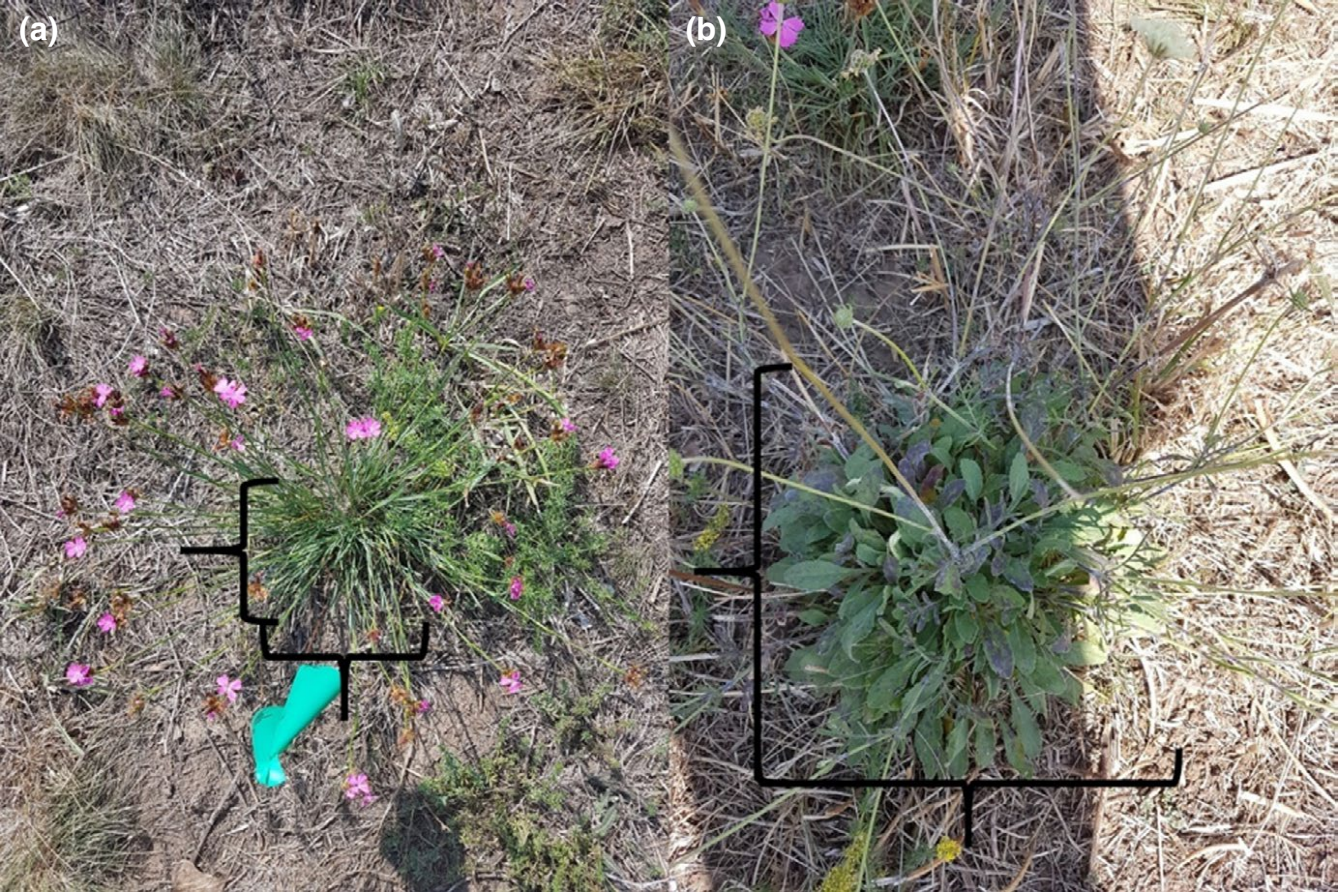
(a) Measurement of basal area for *Dianthus carthusianorum*, the black brackets indicate the way the size was measured. In addition, one of the flags, used to mark the individuals, is visible. (b) Measurement of basal area of *Scabiosa ochroleuca*, black brackets indicate the way size was measured

In late spring/early summer (June 2019), two weeks after the first mowing event, we randomly chose eight individual plants per species in each of the 10 plots (160 different plants: 40 plants for each species and climate treatment combination). The minimum distance between individuals was one meter to avoid selecting clones. We marked selected plants with a flag and colored strings. The flag was used to find the plants quickly, while the colored strings indicated the experimental pollination treatment (black = control, yellow = supplement). As a measure of size, we used the basal area of the plant (length × width) by measuring the longest side of individuals (length) and the longest side perpendicular to the first measurement (width) using a measuring stick (Figure 1). The sizes were afterward analyzed to see whether we by chance chose bigger or smaller individuals in a treatment. *D*. *carthusianorum* did not show differences between treatments in size, but *S*. *ochroleuca* did (Appendix 4). Because we assumed an effect of plant size on intact seeds per reproduction unit (seed heads and seed capsules), we calculated a second model, with plant size as random effect (Appendix 5).

We assigned the eight plants in each plot to one of two pollination treatments (control, supplement). Plants in the control treatment were not manipulated and were open to natural pollination, whereas those in the supplement treatment were open to natural pollination and also received additional pollen via hand pollination. Pollen came from three different plants that were randomly chosen but were at least two meters away from the recipient individual, within the same plot, and contained pollen that was clearly visible on anthers. For *D*.* carthusianorum*, we used three anthers to brush pollen on to the stigma of the flowers of one individual. For *S*.* ochroleuca*, we collected three flower heads and rubbed them on the marked individual's flower head. We applied supplemental pollen every other day to the flowers of both species throughout the flowering period.

We compared the amount of flowers between the climate treatments in each month and calculated the percentage of individuals that flowered each month (Appendix 6). We did not find any evidence that would let us assume a direct effect of flowering timing or number of flowers on the foraging decisions of pollinators.

### 2.4 Data collection

**FIGURE 2:**
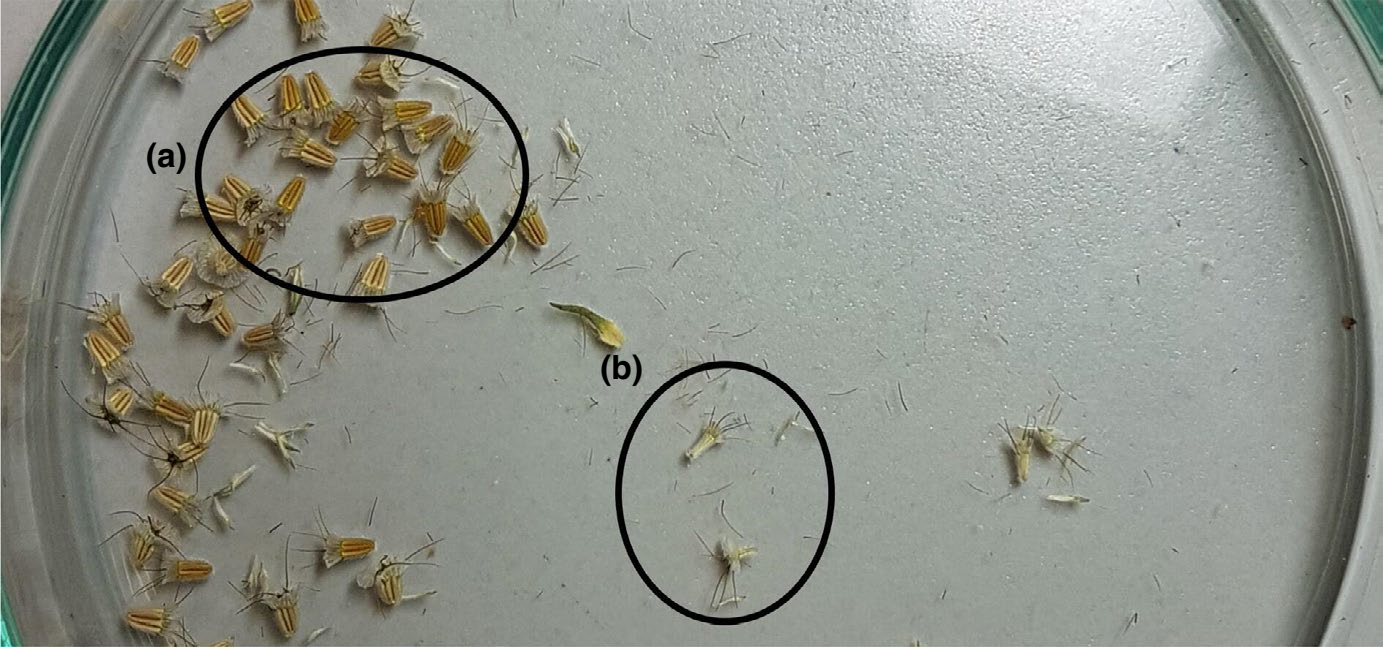
Seeds of *Scabiosa ochroleuca* that we considered to be (a) intact seeds and (b) non‐intact

We collected mature seed capsules (*D*. *carthusianorum*) and seed heads (*S*.* ochroleuca*), placed them in paper bags, and stored these bags in a cold chamber at the German Centre for Integrative Biodiversity Research (iDiv) in Leipzig. We recorded information about whether seed capsules of *D*. *carthusianorum* were closed, half‐open (if the capsule showed a little opening), or open (if the capsule opened in the field before collection) at the time of collection. We excluded seed capsules that were open at time of collection from the statistical analysis, as we could not guarantee that we did not lose seeds in the field. We checked individual plants every other day and harvested mature seed capsules and seed heads. Later, we counted the number of intact‐ and non‐intact seeds. Seed viability was based on seed size and color (Figure 2).

### 2.5 Statistical analysis

Intact seeds per capsule/head for each individual and season are the sum of intact seeds divided by the sum of capsules/head. Our explanatory variables were climate treatment (ambient and future), pollination treatment (control and supplement) and season (summer and fall). Every capsule or head collected before September was considered as summer capsules/heads, as everything after 1st of September was considered as fall offspring, consistent with the change in precipitation regime within the GCEF. Our pollination experiment represents a split‐plot design on its own: The main plots in our design are the extensively used meadow plots, representing the two climate treatments, and the subplots (or sub‐units) are the individuals within a plot, representing the pollination treatments. We conduced separate statistical analysis for each species as they differ in their many important traits, such as size, evolutionary history, and reproductive output, and because we did not have a‐priori hypotheses how and why their responses should differ. To analyze the data, we applied repeated‐measurements linear mixed‐effect models using Proc Mixed in SAS version 9.4 (SAS Institute Inc.) to analyze our data based on a split‐plot repeated‐measure design (as the samples were taken from the same individual in the two different seasons). We included the fixed effects climate, pollination, and season and all possible two‐ and three‐way interactions. As random effects, we included plot nested within climate (error term at the main plot level) and individual nested within pollination x plot x climate (error term at the subplot level) for the between‐subject model, and season x plot nested within climate (error term at the main plot level) for the within‐subject model. Further, as it is possible that an individual's seed production in the summer could influence its seed production in the fall, we tested for temporal autocorrelation. Since the first‐order autoregressive covariance structure turned out to be significant, we incorporated it into the model. In case of significant interactions, these interaction effects are partitioned into simple main effects of each factor at each single level of the other factor (Woodward & Bonett, 1991) using the slice option of the Proc Mixed package in SAS. For visualizing the data, we used Rstudio (R version 4.0.3, R core team, 2020) including the packages ggplot2 (Wickham, 2016) and xlsx (Dragulescu & Arendt, 2020). The SAS code and the R code can be found at GitHub (https://github.com/Martin19910130/_future_pollen_limitation).

## 3 RESULTS

For the analysis of *D*. *carthusianorum*, 1063 seed capsules from 75 individuals (61 summer individuals and 72 fall individuals) were included in the analysis (283 capsules for the summer ambient treatment, 324 for the fall ambient, 235 for the summer future, and 221 for the fall future). For *S*.* ochroleuca*, 1465 seed heads from 65 individuals (50 summer individuals and 64 fall individuals) were included in the analysis (126 seed heads for the summer ambient treatment, 545 for the fall ambient, 171 for the summer future, and 623 for the fall future). Due to herbivory, pathogens, and other factors, we lost five *D*. *carthusianorum* and 16 *S*. *ochroleuca* individuals over the course of the experiment.

**FIGURE 3:**
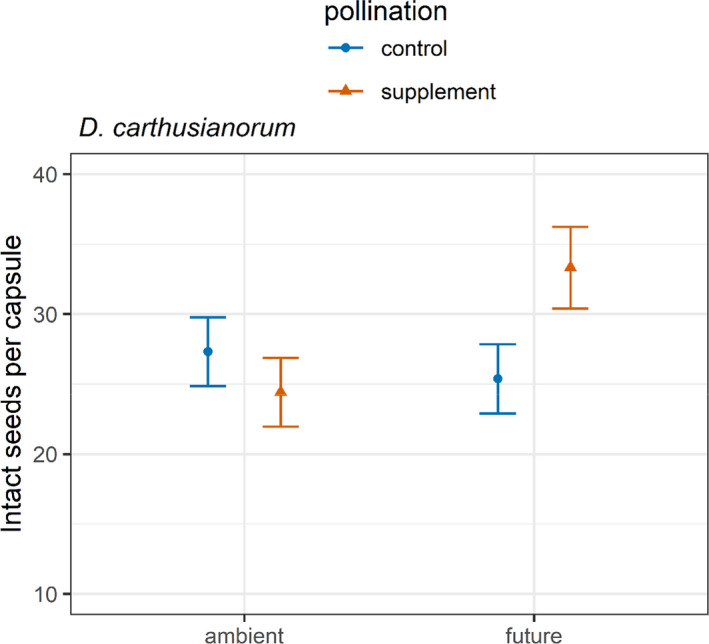
Effect of the interaction between pollen and climate treatments on the number of intact seeds per seed capsule on *Dianthus carthusianorum*. Shown are the mean and the standard errors

We found that the climate and pollination treatments interactively influenced the number of intact seeds per capsule for *D*. *carthusianorum* (Table 1). *Dianthus carthusianorum* produced the highest number of intact seeds when given supplemental pollen in the future climate treatment. Decomposing the significant interaction into the simple main effects of a given factor at each level of the other factor, we found that the pollination treatment had a significant effect on the number of intact seeds only under future climate (*F*_1,63_ = 4.32, *p* < .05) and not under ambient climate conditions (*F*_1,63_ = 0.69, *p* = .41). Accordingly, future climate only affected the number of intact seeds per capsule of *D*. *carthusianorum* under supplemental pollen treatment (*F*_1,63_ = 5.47, *p* < .05). Plants supplemented with pollen, produced 7.95 (31%) more seeds than the control plants under future climate conditions (pollen control, future climate: 25.37 ± 2.46, pollen supplement, future climate: 33.32 ± 2.91; mean ± *SE*) (Figure 3). Additionally, plants in the supplemental pollen treatment produced 8.90 (35%) more seeds in future climate than in the ambient climate treatment (pollen supplement, ambient climate: 24.42 ± 2.46, pollen supplement, future climate: 33.32 ± 2.91) (Figure 3).

**TABLE 1:** Results of the mixed‐effect model with intact seeds per reproductive unit (*Dianthus carthusianorum* = capsule, *Scabiosa ochroleuca* = seed head) as response variable

	*D. carthusianorum*			*S. ochroleuca*		
	*df*	*F*‐value	*p*‐value	*df*	*F*‐value	*p*‐value
Climate	1, 8	1.82	.21	1, 8	1.60	.24
Pollination	1, 63	0.96	.33	1, 53	1.58	.22
Climate × Pollination	1, 63	**4.39**	<.05*	1, 53	1.72	.20
Season	1, 8	**4.17**	**<.1**	1, 8	**5.37**	<.05*
Season × Climate	1, 8	0.98	.35	1, 8	1.33	.28
Season × Pollination	1, 46	0.41	.52	1, 37	0.13	.72
Climate × Season × Pollination	1, 46	0.16	.69	1, 45	0.90	.35

Note

The climate treatment consists of ambient and future, the pollination treatment consists of control and supplemental, and the seasons include summer and fall. *df* refers to the numerator and denominator degrees of freedom. Significant *F*‐ and *p* values are shown in bold: *p* < .1,*p < .05.

**FIGURE 4:**
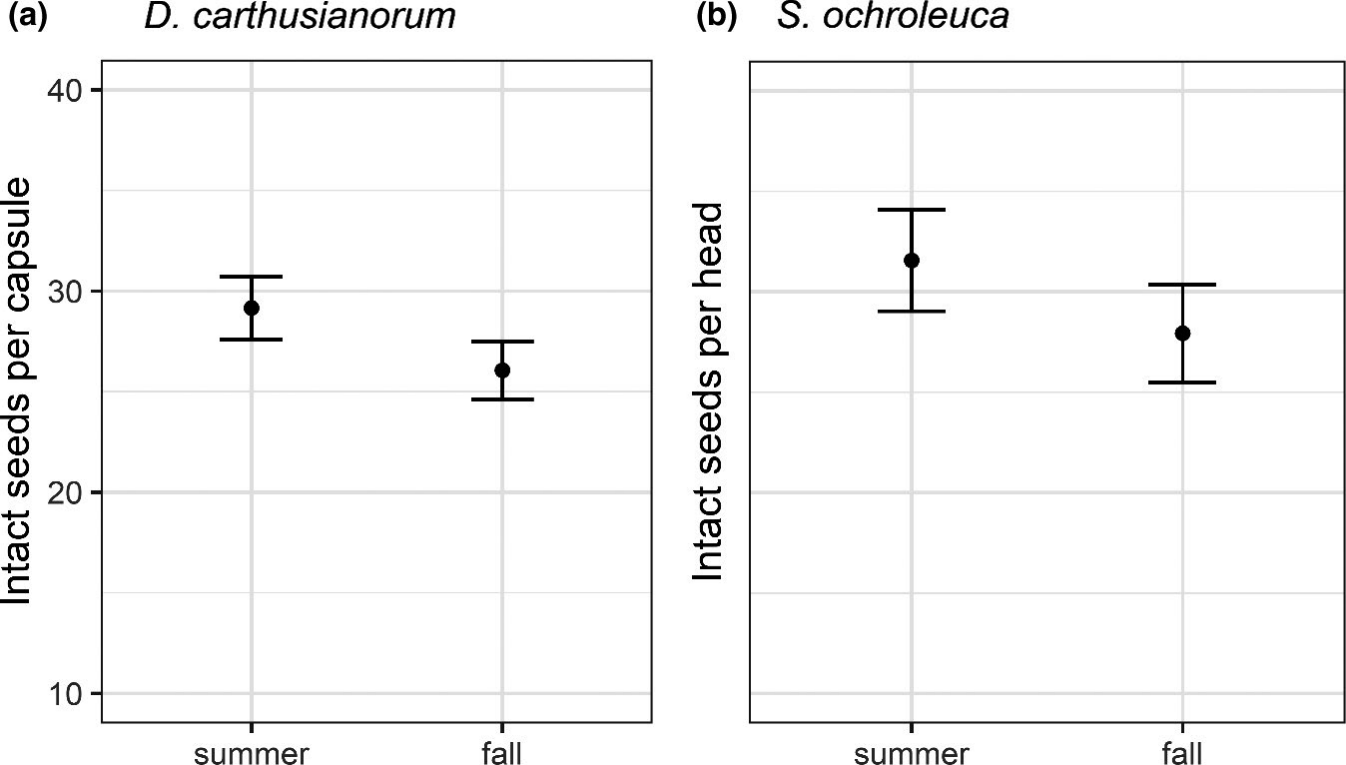
Effect of season (summer and fall) on the number of intact seeds per reproductive unit, shown are the mean and standard error for: (a) *Dianthus carthusianorum* and (b) *Scabiosa ochroleuca*

For both species, we found an effect of season on the number of intact seeds per capsule/head; for *S*.* ochroleuca*, this effect was significant (Table 1), and for *D*.* carthusianorum*, this effect was marginally significant (Table 1). *Scabiosa ochroleuca* produced 3.63 seeds per head more in summer than in fall, which is an increase of 13% (summer: 31.55 ± 2.52, fall: 27.92 ± 2.43) (Figure 4). *D*. *carthusianorum* produced 12% more seeds (3.1 intact seeds more per capsule) in summer than in fall (summer: 29.15 ± 1.56, fall: 26.05 ± 1.43) (Figure 4). If we included size as a random variable in our models, the significant effect of season on the number of intact seeds per head in *S*. *ochroleuca* was slightly reduced and became marginally significant (Appendix 5), indicating that the increased number of intact seeds was partly mediated by an increase in plant size.

## 4 DISCUSSION

We hypothesized that pollination, climate, and season would interactively influence the reproductive success of our focal pollinator‐dependent grassland species and specifically that the effects of the pollen supplementation treatment would be most dramatic in the future fall treatment combination for which water availability is highest. We did not find support for this rather specific hypothesis, but instead found other main and interactive effects of our treatments on *D*. *carthusianorum* and *S*. *ochroleuca*.

Both plant species had higher reproductive success in the summer season independent of the pollination treatment, although the result for *D*. *carthusianorum* was only marginally significant. The early mowing that occurs in our study system likely increased the reproductive success of these late‐flowering species, because they are able to quickly re‐sprout after early season mowing events and then grow without much competition (Klotz et al., 2002). This result is similar to those found in other studies (Brys et al., 2004; Endels et al., 2007; Nakahama et al., 2016). For example, Nakahama et al. (2016) showed that early mowing increased inflorescence and the fruit production of *Vincetoxicum pycnostelma Kitag*, whereas mowing later in the season had negative effects. The high reproductive success of our focal species in the summer season can also be attributed to their drought tolerance (Klotz et al., 2002; Zalłçcka & Wierzbicka, 2002). Drought‐tolerant species are known to be more affected by competition than water availability in grassland habitats (Kardol et al., 2010). The year we conducted this study (2019) was considered a drought year in our region, and our focal species might have benefited from the especially low biomass of their competitors during the summer (H. Auge, unpublished data). Better pollinator services in the summer compared with the fall (e.g., due to higher insect activity) are unlikely to explain our results, as we did not find any differences in the pollination treatments in pollen limitation between seasons. If insects’ behavior coursed this finding, we would expect an increase in the supplemental pollen treatment compared with the control treatment as it should be independent of insect activity and behavior.

Pollination supplementation did not show any main or interactive effect on the reproductive success of *S*.* ochroleuca*, indicating that this species is currently not pollen limited and is not expected to become pollen limited with climate change. This result should be interpreted with caution, because it might result from the dry summer conditions and low competition across all of our treatments in the year of this experimental study and/or from the excellent landscape context our experiment in embedded in. Our focal plant species were some of the most dominant flowering plant species in the site in our study year and were thus clearly visible to pollinators. Co‐flowering plants are known to sometimes compete for the services of pollinators (Levin & Anderson, 1970; Sargent & Ackerly, 2008), and this competition might have also been lower in our study year. Both of our focal species are highly generalized in their pollination, attracting several species of bees, flies, and butterflies (Klotz et al., 2002). Thus, in years with more dominance of co‐flowering plant species, heterospecific pollen receipt might reduce reproductive success (Ashman et al., 2020; Arceo‐Gómez et al., 2019). Further, we note that our study area is embedded within a large field site of managed grasslands. These diverse, well‐managed meadows create an environment that likely supports a high abundance and diversity of pollinators our study plots might draw in insects from the surrounding landscape that provide excellent pollination services. Climate change treatments in other landscape contexts might have more negative consequences on the reproductive success of *S*. *ochroleuca*.

*Dianthus carthusianorum* has higher reproductive output in future climate conditions, but only when given supplemental pollen. Thus, in these grasslands, the lower summer and higher fall precipitation conditions that are projected to occur in the future with climate change have the potential to increase the reproductive fitness of this plant species, but only if pollination services also improve. Currently, pollination services are inadequate to fully pollinate this plant species in the experimental future climate conditions. There are many possible mechanisms that could explain this. The primary pollinators of *D*. *carthusianorum* are bees and butterflies (Bloch et al., 2006). The abundances of these pollinators might not be high enough to provide adequate services to *D*. *carthusianorum* in the future climate treatment. Alternatively, these pollinators might avoid foraging in future climate plots, or preferential visit plants other than *D*. *carthusianorum*, due to the altered composition of floral resources in this treatment. In our experiment, we did not find evidence for shifting phenologies of the focal plant species between the ambient and future climate treatments, and thus, temporal mismatches are not responsible for pollen limitation in the future climate treatment. However, in the context of future climate change, insect phenologies can also change, and temporal mismatches may occur. Climate change is known to cause temporal mismatches between plants and pollinators due to shifting phenology (Fitter & Fitter, 2002; Gordo & Sanz, 2010), which can cause pollen limitation (Kudo & Cooper, 2019).

We suggest that future research should expand our study to additional plant species, to larger experimental approaches, and should measure additional response variables. Our research focused on plant species that are rather generalized in their pollination, which is typical (and therefore representative) for European grasslands (Herrera, 1996; Olesen, 2000). However, specialized plant species are more commonly pollen limited (Bennett et al., 2020; Knight et al., 2005; Martén‐Rodríguez & Fenster, 2010) and might be more sensitive to climate change under future climate conditions. While our experimental climate treatment plots are large in size compared with plots in other global change experiments, pollinators can still easily foraging between plots in different experimental treatments (Chapman et al., 2003). Creating experiments in which pollinators respond to climate treatments in their abundances and behavior is difficult, but could involve measuring pollen limitation across different years or across large spatial gradients that naturally differ in their climate, or by creating larger field or greenhouse experiments, across landscapes, that allowed pollinators to exhibit natural foraging behavior and have appropriate nesting habitats. We suggest that future research should measure offspring quality in addition to offspring quantity as quality can also change with resource availability and pollination regime (Bommarco et al., 2012).

In conclusion, we showed that climate change does not negatively influence plant reproductive success of our two grassland plant species, and reproductive success of one of our focal plant species could even improve under future climate if pollinator services also improved. Our study is the first to detect pollen limitation under a realistic climate change scenario. We contribute toward developing a general understanding of how resources and pollen addition interactively influence plant reproduction. However, whether this difference is due to pollen quantity or quality cannot be determined by our experimental approach. It is known that not only the amount of pollen can alter reproduction success but also the quality of the pollen. Considering the importance of plant reproduction for the development of plant populations (Jacquemyn et al., 2010), we hope that our study will help motivate future research considering pollen limitation in the context of climate change.

## CONFLICT OF INTEREST

No author has a conflict of interest.

## AUTHOR CONTRIBUTIONS

**Martin Andrzejak:** Conceptualization (supporting); Formal analysis (lead); Methodology (lead); Software (lead); Writing – original draft (lead); Writing – review & editing (lead). **Lotte Korell:** Conceptualization (equal); Methodology (equal); Supervision (supporting); Writing – original draft (supporting); Writing – review & editing (equal). **Harald Auge:** Formal analysis (supporting); Software (supporting); Writing – review & editing (supporting). **Tiffany M. Knight:** Conceptualization (lead); Methodology (supporting); Supervision (lead); Writing – original draft (supporting); Writing – review & editing (supporting).

## Figures and Tables

**FIGURE A1:**
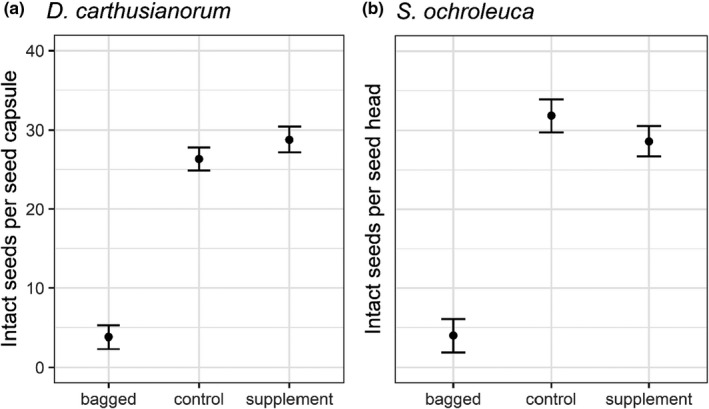
Effect of the pollination treatment (bagged, control, and supplement) on the intact seed per reproductive unit, shown are the mean and standard error for: (a) *Dianthus carthusianorum* and (b) *Scabiosa ochroleuca*. *The difference between supplement and control is not significant in both species*

**FIGURE A2:**
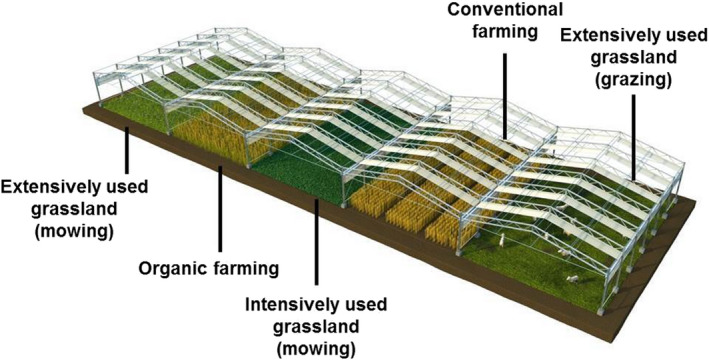
Layout of a GCEF main plot which is divided into 5 different land‐use plots. (Figure: Tricklabor/Marc Hermann)

**FIGURE A3:**
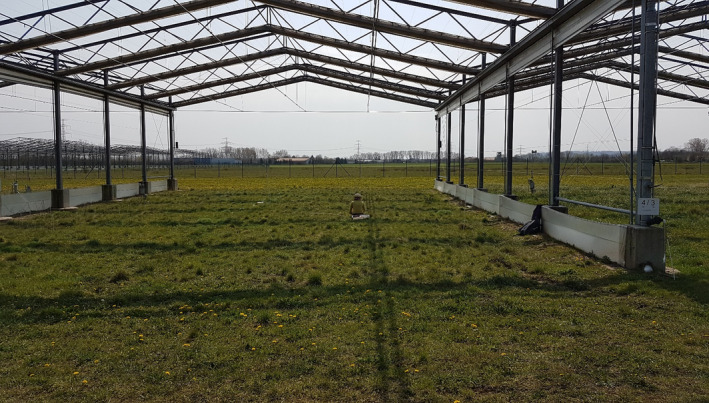
Extensively used meadow sometime after the mowing event. Seen is the steel construction with the sides and the roofs open

**FIGURE A4:**
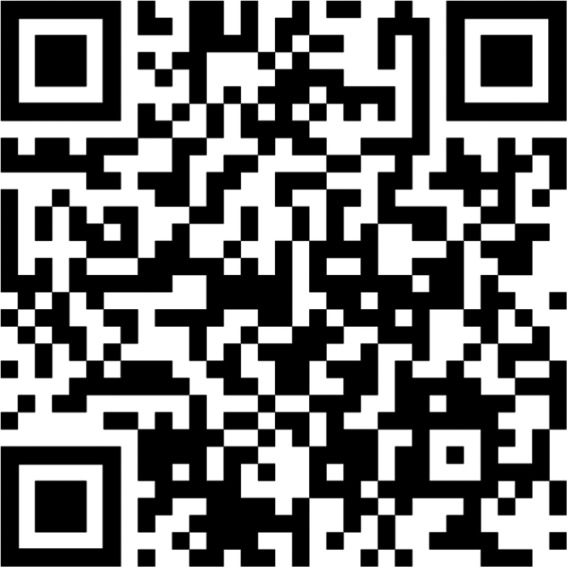
QR code to GitHub page of this project

## Data Availability

Data (original and mean intact seeds per capsule/head, including plant size): https://figshare.com/projects/Pollen_supplementation_experimant/122333
*Scabiosa ochroleuca* mean intact seeds per head and season: 10.6084/m9.figshare.16577774.
*Dianthus carthusianorum* mean intact seeds per capsule and season: 10.6084/m9.figshare.16577771
*Scabiosa ochroleuca* original data: 10.6084/m9.figshare.16577777
*Dianthus carthusianorum* original data: 10.6084/m9.figshare.16577768 The code used will be accessible on GitHub.

## APPENDIX 1

### Methods for bagged treatment

To test the autonomous selfing ability of *Dianthus carthusianorum* and *Scabiosa ochroleuca*, we established a pollinator exclusion experiment. We randomly chose four plants on each of the 10 plots with a minimum distance of one meter between all other experimental individuals. We bagged flower buds of those plants with organza bags in order to make them inaccessible for insects to pollinate. Similar to the supplemental pollen experiment, we checked on the individuals every other day and bagged new buds, as they appeared. If we missed a bud and it developed into a flower, we did not include that flower in the study. During the course of the pollinator exclusion experiment, ants and caterpillars found their way into the organza bags eventually, maybe providing very little pollen. At very windy days, some bags flew off of the flowers or buds. We also excluded those flowers in the analysis.

Similar to the other pollination treatments, we counted the number of intact and non‐intact seeds. Seed viability was based on seed size and color. To calculate statistical differences between treatments, the same mixed models with the same parameters where used.

## APPENDIX 2

The results of the pollinator exclusion experiment show that there is a significant difference between the pollination treatments in both species (Table A1). The bagged treatment did not produce even half of the intact seeds that the other two treatments were able to produce (Figure A1). If we compare the *D*. *carthusianorum* bagged treatment to the control, the bagged treatment only produces 14.43% of the intact seeds that the control treatment produces. Comparing bagged treatment to supplemental pollination, only 13.20% intact seeds are produced in the bagged treatment (bagged = 3.80 ± 1.47, control = 26.34 ± 1.46, supplement = 28.78 ±1.6). *Scabiosa ochroleuca* shows a similar pattern, bagged only produces 12.51% of intact seeds in the bagged treatment compared with the control treatment, while the supplemental treatment produced around 86.09 more intact seeds compared with the bagged plants (bagged = 3.98 ± 2.08, control = 31.82 ± 2.06, supplement = 28.61 ± 1.9).

**TABLE A1:** Results of the mixed effect model with intact seeds per reproductive unit (*Dianthus carthusianorum* = capsule, *Scabiosa ochroleuca* = seed head) as response variable

	*D. carthusianorum*			*S. ochroleuca*		
	*df*	*F*‐value	*p*‐value	*df*	*F*‐value	*p*‐value
Climate	1, 8	1.80	.22	8	0.49	.50
Pollination	2, 100	**84.95**	**<.0001*****	1, 85	**83.00**	**<.0001*****
Climate × Pollination	2, 100	**3.23**	**<.05***	2, 85	**4.20**	**<.05***
Season	1, 8	**4.50**	**.07**.	1, 8	**5.65**	**<.05***
Season × Climate	1, 8	0.06	.82	2, 8	**3.72**	**.09**.
Season × Pollination	2, 75	0.49	.62	2, 50	0.17	.85
Climate × Season × Pollination	2, 69	1.12	.33	2, 50	0.83	.44

Note

The climate treatment consists of ambient and future, the pollination treatment consists of control, supplemental, and bagged, and the seasons include summer and fall. *df* refers to the numerator and denominator degrees of freedom. Significant *F*‐ and *p* values are shown in bold: *p* < .1,**p* < .05, ***p* < .01, ****p* < .001.

## APPENDIX 3

Shown is the general layout of the GCEF, including all five land‐use treatments this experiment was only conducted on the extensively used grassland (Figure A2). The photograph shows one of our extensively used meadows after the mowing event (Figure A3).

## APPENDIX 4

We compared the plant size between treatments to ensure that we did not, by chance chose bigger individuals in the treatments which could have influenced the intact seeds per reproductive unit. To do so, we log‐transformed the size data.

*Dianthus carthusianorum* did not show any significant difference in size between the treatments in both seasons (Table A2), while *Scabiosa ochroleuca* showed in both seasons a significant difference in size between the climate treatments (Table A3).

**TABLE A2:** Results of the mixed effect model for *Dianthus carthusianorum* with plant size as dependent variable

*Dianthus carthusianorum*						
	Summer			Fall		
	*df*	*F*‐value	*p*‐value	*df*	*F*‐value	*p*‐value
Climate	1, 8	0.91	.37	1, 8	0.12	.74
Pollination	1, 49	0.21	.65	1, 60	0.39	.53
Climate × Pollination	1, 49	0.17	.68	1, 60	0.72	.40

Note

Climate consists of ambient and future while pollination includes the treatments control and supplement. *df* refers to the numerator and denominator degrees of freedom. Significant *F*‐ and *p* values are shown in bold: *p* < .1,**p* < .05, ***p* < .01, ****p* < .001.

**TABLE A3:** Results of the mixed effect model for *Scabiosa ochroleuca* with plant size as dependent variable

*Scabiosa ochroleuca*						
	Summer			Fall		
	*df*	*F*‐value	*p*‐value	*df*	*F*‐value	*p*‐value
Climate	1, 8	**6.48**	**<.05***	1, 8	**13.98**	**<.01****
Pollination	1, 38	0.18	.68	1, 52	0.18	.67
Climate x Pollination	1, 38	0.01	.92	1, 52	1.39	.24

Note

Climate consists of ambient and future while pollination includes the treatments control and supplement. *df* refers to the numerator and denominator degrees of freedom. Significant *F*‐ and *p* values are shown in bold: *p* < .1,**p* < .05, ***p* < .01, ****p* < .001.

## APPENDIX 5

In order to understand how size influences the intact seed output of both species, we decided to include it as random variable into our model, to make sure we did not measure any indirect effects of plant size. *D*. *carthusianorum* did not show any difference in the significance levels compared with the models without size (Table A4).

**TABLE A4:** Results of the mixed effect model with intact seeds per reproductive unit (*Dianthus carthusianorum* = capsule, *Scabiosa ochroleuca* = seed head) as response variable

	*Dianthus carthusianorum*			*Scabiosa ochroleuca*		
	*df*	*F*‐value	*p*‐value	*df*	*F*‐value	*p*‐value
Climate	1, 8	1.82	.21	1, 8	1.42	.26
Pollination	1, 62	0.96	.33	1, 52	1.68	.20
Climate × Pollination	1, 62	**4.39**	**<.05***	1, 52	1.58	.21
Season	1, 8	**4.17**	**<.1**.	1, 8	**5.26**	**<.1**.
Season × Climate	1, 8	0.98	.35	1, 8	1.31	.29
Season × Pollination	1, 46	0.41	.52	1, 37	0.12	.73
Season × Climate × Pollination	1,46	0.16	.69	1, 37	0.89	.35

Note

The climate treatment consists of ambient and future, the pollination treatment consists of control and supplemental, and the seasons include summer and fall. This model includes plant size as an additional random variable. *df* refers to the numerator and denominator degrees of freedom. Significant *F*‐ and *p* values are shown in bold: *p* < .1,**p* < .05, ***p* < .01, ****p* < .001.

For *S*. *ochroleuca,* the significant effect of season on the reproductive success was reduced to a marginal significant effect (Table A4). Indicating that the increased number of intact seeds per seed head under future climate conditions was partly mediated by an increased plant size.

## APPENDIX 6

To understand whether insect behavior and foraging decisions are influenced by flowering phenology, we gathered information about the flowering individuals and the flower count of them. The idea was that individuals in the different climate treatments flower at different times, providing insects different resources for pollination. For example, individuals grown in the future climate could flower earlier mediated by the warming and precipitation differences. This could influence our pollination treatments. We used repeated‐measures linear mixed‐effects models to analyze whether the mean number of flowers per individual for each plot and month depended on climate, month, and their interaction (fixed effects). Our response variable was log‐transformed after adding 0.1 (to include zero values) because positive skewness of residuals suggested a lognormal distribution. A repeated‐measurement model was used. A first‐order autoregressive covariance structure improved the model fit just for *Scabiosa ochroleuca*.

We also considered the probability of flowering of the individuals for each month as the response variable and used a repeated‐measures generalized linear mixed‐effects model with similar model structure like for the mean number of flowers but with binomial distribution of residuals and logit‐link function. A first‐order autoregressive covariance structure improved the model fit for both study species.

We did not find evidence for different flower timings between the individuals grown in ambient vs future climate for *Dianthus carthusianorum* and *Scabiosa ochroleuca*, meaning no difference between mean number of flowers (Table A5 ) or probability of flowering (Table A6) was found with respect to the climate treatment. Only significant differences in the response variables were found between the different months, which was to be expected. This means that the phenology of both study species was not changed by the climate treatment in the GCEF.

**TABLE A5:** Results of the mixed effect model with mean number of flowers as response variable

	*Dianthus carthusianorum*			*Scabiosa ochroleuca*		
	*df*	*F*‐value	*p*‐value	*df*	*F*‐value	*p*‐value
Climate	1, 8	0.29	.61	1,8	1.49	.26
Month	5, 40	**2.98**	**<.05***	5,40	**15.07**	**<.001*****
Climate × month	5, 40	1.08	.39	5,40	0.53	.75

Note

The climate treatment consists of the two levels ambient and future, and the month included in this model is as follows: June, July, August, September, October, and November. *D*.*f*. refers to the numerator and denominator degrees of freedom. Significant *F*‐ and *p* values are shown in bold: *p* < .1,**p* < .05, ***p* < .01, ****p* < .001.

**TABLE A6:** Results of the mixed effect model flower probability of the 8 individuals as response variable

	*Dianthus carthusianorum*			*Scabiosa ochroleuca*		
	*df*	*F*‐value	*p*‐value	*df*	*F*‐value	*p*‐value
Climate	1, 8	0.00	.95	1, 8	0.82	.39
Month	5, 40	**19.22**	**<.001*****	5, 40	**12.42**	**<.001*****
Climate × month	5, 40	1.75	.15	5, 40	0.36	.87

Note

The climate treatment consists of the two levels ambient and future, and the month included in this model is as follows: June, July, August, September, October, and November. *df* refers to the numerator and denominator degrees of freedom. Significant *F*‐ and *p* values are shown in bold: *p* < .1,**p* < .05, ***p* < .01, ****p* < .001.

QR code directing to the GitHub page of this project, where you can find the SAS and R code as well as the data of this research project (Figure A4).
